# External morphology of the mouthparts and observations on behavior of *Tuckerella japonica* on *Camellia sinensis* in the continental USA

**DOI:** 10.1007/s10493-017-0204-0

**Published:** 2017-12-18

**Authors:** Carl C. Childers, Enrico de Lillo, Gary R. Bauchan, Michael E. Rogers, Ronald Ochoa, Christopher Robinson

**Affiliations:** 1 Citrus Research and Education Center, University of Florida, 700 Experiment Station Road, Lake Alfred, FL 33850 USA; 2 Hendersonville, NC 28792 USA; 3 Dipartimento di Scienze del Suolo, della Pianta e degli Alimenti, University of Bari ‘Aldo Moro’, via Amendola, 165/a, 70126 Bari, Italy; 4 Electron and Confocal Microscopy Unit (E&CMU), USDA-ARS, 10300 Baltimore Avenue, BARC-West, Beltsville, MD 20705 USA; 5 Systematic Entomology Laboratory, USDA-ARS, 10300 Baltimore Avenue, BARC-West, Beltsville, MD 20705 USA; 6 Summerville, SC 29485 USA

**Keywords:** Tetranychoidea, Plant feeders, Gnathosoma, Functional morphology, Ethology

## Abstract

*Tuckerella japonica* Ehara (Acari: Tetranychoidea: Tuckerellidae) was found on stems of seedling plants of *Camellia sinensis* (L.) O. Kuntze (Theaceae) in the continental USA. This mite is able to pierce exposed green periderm tissue with its paired stylets on 1- to 3- or 4-year-old stems where the outer bark had split longitudinally. The mite was not found on branches older than 3 or 4 years, where splitting diminished and, eventually, a uniform covering of bark was formed. The mouthparts of *T. japonica* were examined under scanning electron microscopy and their external morphology was compared with known Tetranychoidea. There were usually one or two feeding holes in an area where the female subsequently deposited one or more eggs. Females were observed defending the areas where their eggs and/or young occurred. This behavior along with potentially limited access to exposed periderm or availability of shoots in the rows of plants may restrict higher populations of *T. japonica* from becoming established. The potential of *T. japonica* as an economic pest remains unknown at this time.

**Electronic supplementary material:**

The online version of this article (10.1007/s10493-017-0204-0) contains supplementary material, which is available to authorized users.

## Introduction

The family Tuckerellidae is one of five families within the superfamily Tetranychoidea that also includes the spider mites (Tetranychidae), false spider mites (Tenuipalpidae), Allochaetophoridae and Linotetranidae (Smith Meyer and Ueckermann 1997). Mites of these five families are obligatively phytophagous.

The family Tuckerellidae consists of a single genus, *Tuckerella* Womersley, and a key to species was provided by Smith Meyer and Ueckermann (1997). Twenty-eight species were reported by Zhang et al. (2011) whereas 29 species were listed by Beard and Ochoa (2010). Increased interest in tuckerellid mites has evolved given their presence on fruits, leaves and stems of imported plants (Beard et al. 2013). Charles (2009) reviewed the tuckerellid species of the world and reported their occurrence on woody parts of plants including stems and fruit. He aptly stated that no definitive information was known on feeding or their economic importance.

The only species of *Tuckerella* mentioned by Jeppson et al. (1975) was *Tuckerella pavoniformis* (Ewing). No mention was made of its pest status with respect to the host plants that were listed: citrus, hibiscus, papaya, tea and various non-economical plants. *Tuckerella pavoniformis* was reported in association with witches’ broom on *Ligustrum japonicum* Thunberg (Oleaceae) and other unidentified plant species in Florida (Welbourn 1998).

Corpus-Raros (1989) found *Tuckerella ornata* (Tucker) within protected cracks or beneath scales on guava plant stems. One female *Tuckerella japonica* Ehara was collected from a twig of *Cryptomeria japonica* (Thunberg ex. L.f.) D. Don (Cupressaceae) in New Zealand and reared successfully on the sepal end of a kiwifruit, *Actinidia deliciosa* (A. Chev.) C. F. Lang and A. R. Ferguson (Actinidiaceae) (Zhang and Henderson 2012). No mention was given if the female successfully oviposited or produced young.

*Tuckerella kumaoensis* Gupta was reported to be a pest of Sapota, *Manilkara achras* Mills Foseberg (Sapotaceae) in India by Shukla et al. (2013). *Manilkara achras* is a synonym of *Manilkara zapota* (L.) P. Royen (International Plant Names Index 2012). The authors observed the mite feeding on immature marble-sized fruit but failed to clarify whether they continued feeding on the fruit to maturity. No mention was made on the distribution of tuckerellid mites or their eggs on other Sapota plant parts.

Several species of *Brevipalpus* (Acari: Tenuipalpidae) feed on leaves, stems and fruit of plants, including citrus, passion fruit, coffee, and many ornamental species (Chagas et al. 2003; Childers et al. 2003; Kitajima et al. 2003, 2014). The cytoplasmic and nuclear types of citrus leprosis virus complexes are non-systemic and are vectored by two or more *Brevipalpus* species (Rodrigues et al. 2003; Kitajima et al. 2014; Sanchez-Velazquez et al. 2015). In many instances, lesions from these non-systemic viruses occurred on stems as well as on leaves and fruit, thus indicating that these mites fed on stems too.

Childers et al. (2016) found *T. japonica* on the green periderm tissue that becomes exposed when the bark splits on stems of *C. sinensis*. Plants produced from both seed or unknown tea cultivars were included in the study. Significantly greater numbers of *T. japonica* were found on the periderm of 2-year-old stems versus 1- or 3+-year-old stems and were rarely found on leaves. Mites fed on the periderm and underlying cortical tissues as well as on maturing green seed pods of the tea plants (Childers et al. 2016; Achor et al. 2017). No studies show how and where tuckerellid species feed on their respective host plants. Reports of their being economic pests in various crops are questionable as no definitive research has demonstrated these important points.

The outer surface of plants is covered by the dermal tissue system, which includes the epidermis, the primary outer protective covering of the plant body. The periderm is the protective tissue or covering of the plant body of secondary origin. The periderm supplants the epidermis, in plants that undergo a secondary increase in thickness (Evert 2006).

Three layers of woody or corky tissue occur on stems of some plants including seedlings of *Camellia sinensis* (L.) O. Kuntze (Theaceae). The outer layer is the phellem that includes crevices and dead plant cells, the middle layer is the phellogen that includes the periderm meristem (= green stem tissue), and the inner layer is the phelloderm, mostly composed of parenchyma, which takes the place of the cortex or cortical cells in other plants or plant parts (Esau 1965). Eames (1936) identified three vegetative plant organs: stem, leaf, and root. The stem and leaf are commonly treated together as a morphological and functional unit and referred to as the shoot (Evert 2006).

The feeding habit and mouthpart structures of tuckerellids appear to be quite similar to other tetranychids. The functional morphology of the gnathosoma of some tetranychids and tenuipalpids has been studied, but no data are available on tuckerellids (Lindquist 1985; Alberti and Crooker 1985; Nuzzaci and de Lillo 1989, 1991a, b). A pre-oral food channel and an inter-cheliceral canaliculus were identified in both taxa. The pre-oral food channel is anterior to the labral base. It is composed ventrally of the pre-oral groove, with annular sclerotized reinforcement, and dorsally of the labrum which is interlocked with paired soft and elevated bars of the pre-oral groove (Alberti and Kitajima 2014). The tight connection between the pre-oral groove and labrum hermetically seals the pre-oral food channel which leads to the pharynx via the mouth, located underneath the base of the labrum (Alberti and Kitajima 2014). A small single pore, the rostral fossette, is on the ventral surface of the infracapitulum, and it is connected to the pharynx by the inferior oral commissure. Pre-oral food channel, mouth and pharynx are involved in sucking liquefied food up to the gut, whereas the inferior oral commissure acts as a decompression channel during feeding (Nuzzaci and de Lillo 1989, 1991a). An inter-cheliceral canaliculus is formed by the interlocked stylet-like movable digits when protracted and receives the saliva of the paired salivary gland ducts through the median salivary channel (Nuzzaci and de Lillo 1989, 1991a; Beard et al. 2012; Alberti and Kitajima 2014). These data on tenuipalpids and tetranychids can sustain the description of the external mouthpart structures of *T. japonica* and explain their comparative function.

In this paper, we present information on: (1) external morphology of the mouthparts of *T. japonica* compared to the Tetranychidae and Tenuipalpidae, (2) feeding behavior of *T. japonica* on exposed periderm tissues of *C. sinensis* stems, (3) territorial behavior and distribution of *T. japonica* on *C. sinensis* plants, (4) potential of *T. japonica* as a pest of *C. sinensis* plants, and (5) sampling for tuckerellid mites.

## Materials and methods

### Low temperature scanning electron microscopy (LTSEM) study of the external mouthparts of *Tuckerella japonica*

Two blocks of *C. sinensis* plantings at the Charleston Tea Plantation on Wadmalaw Island in Charleston, SC, USA, were sampled on 21 September 2015 and on 25 May 2016. Approximately 80–100 2-year-old shoots ranging in length from 0.25 to 0.5 m were cut with pruning shears, leaves removed and immediately placed in grocery bags. The samples were taken to the Electron and Confocal Microscopy Unit (E&CMU), USDA-ARS in Beltsville, MD, USA, and kept cool in transit with ice packs in coolers. Samples were processed the following morning on both dates at E&CMU. Each stem was examined for presence of *T. japonica* using a stereomicroscope with a cold light source. Live *T. japonica* motile stages collected in South Carolina were prepared for LTSEM according to Bolton et al. (2014).

### Feeding behavior of *Tuckerella japonica* on exposed periderm tissues of *Camellia sinensis*

Approximately 30–100 2-year-old stems of *C. sinensis* between 0.5 and 1.0 m and with longitudinal splitting of the bark were cut in the field with pruning shears between 2014 and 2016 from the Charleston Tea Plantation on Wadmalaw Island in Charleston, SC, USA. The leaves were removed, returned to the USDA laboratory in Charleston in a cooler and examined using a stereomicroscope with a cold light source. At least 20 of these stems showing evidence of *T. japonica* resting or feeding were selected and 2- to 3-cm-long pieces were cut using pruning shears. A second series of 2- to 3-cm lengths of the same stems with no evidence of mite presence were taken each time for comparison.

Each piece was subsequently cut down the middle of the stem with a razor blade so that the rounded area where the mite(s) were located was evident. Each piece was immediately transferred into one of two vials (mites either present or absent) containing 3% glutaraldehyde in 0.1 M Sorenson’s buffer, pH 7.2, and kept on ice in transit from Charleston until further processing 24 h later in the laboratory at the University of Florida, Citrus Research and Education Center (CREC) in Lake Alfred, FL, USA. There the samples were washed 3× in Sorenson’s buffer, post fixed in 2% osmium tetroxide in the same buffer and kept overnight at 4 °C. The following morning the samples were rinsed again in buffer, dehydrated in ethanol series (10% steps, 10 min for each step) and dried using a Ladd Critical Point Dryer (Ladd Research, Burlington, VT, USA). The dried samples were mounted on stubs, coated with gold/palladium using a Ladd Sputter Coater (Ladd Research) studied and photographed with a Hitachi S530 Scanning Electron Microscope (Hitachi High-Technologies, Japan).

### Distribution and behavior of *Tuckerella japonica* and their progeny on stems of *Camellia sinensis*

Fifty or more samples of 1- to 3+-year-old *C. sinensis* shoots were collected each of 6× between 12 May 2014 and 21 September 2016 from one or two blocks of tea at the Charleston Tea Plantation. Lengths of shoots between 0.25 and 0.5 m were cut in the field with pruning shears, leaves removed and placed in coolers with or without ice depending on temperature. The samples were taken to the USDA Vegetable Laboratory in Charleston for processing. Shoots were examined in the laboratory using a stereomicroscope with a cold light source to observe the behavior and activities of *T. japonica*.

### Color videos of *Tuckerella japonica* behavior

Color images and videos of specimens in situ were obtained using a Hirox KH-7700 Digital Microscope (Hackensack, NJ, USA) with a MXG-5040RZ lens to assess behavior of *T. japonica* motile stages during 2015–2016 at the Electron and Confocal Microscopy Unit in Beltsville, Maryland, USA. The digital microscope possessed focus-stacked capability at 1600 × 1200 pixels per frame with 360° surface imaging and 50–400× zoom magnification. Two videos of female and immature interactions were recorded at 800 × 600 pixels per frame and 15 frames per s.

### Macrophotography of the spatial distribution and aggregation of *Tuckerella japonica* motile and egg stages on stems of *Camellia japonica*

At 08:00 h on 21 September 2016, shoots of *C. sinensis* were collected as described above from *T. japonica* infested areas at the Charleston Tea Plantation and transported to the USDA-ARS Vegetable Laboratory in Charleston. Each stem was examined for presence and behavior of *T. japonica* using a stereomicroscope and a cold light source. A period of 2 h between collection and photography accurately captured the distribution of *T. japonica* life stages on exposed periderm. Little movement by the mites occurred on the stems prior to being photographed. Photographs showing examples of the distribution of the different life stages of *T. japonica* on areas of exposed periderm tissue were obtained using an AxioCam MRC5 camera mounted on a Zeiss Axio Zoom V16 microscope.

The time lag between field collection and photography hindered documenting the original distribution of *T. japonica* on the stems when samples were kept longer than 4 h. Considerable movement of all motile stages on the stem surfaces was observed following these multi-hour delays. We were unable to get the plant samples processed quickly enough at times due to high temperatures in the field or delays in transit due to traffic or weather conditions.

## Results and discussion

**Fig. 1:**
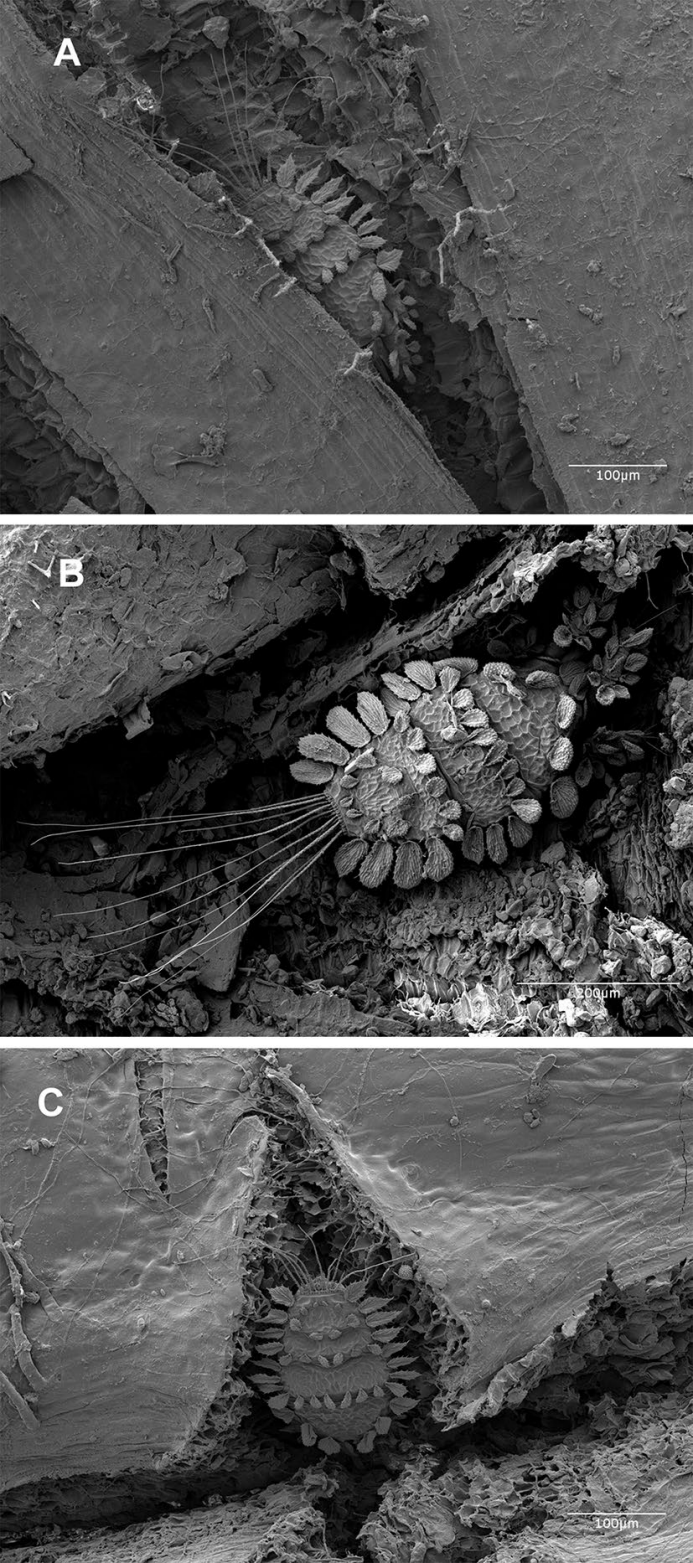
*Tuckerella japonica* frequently concealed in protected crevices or areas created by bark splitting on stems of *Camellia sinensis*

**Fig. 2:**
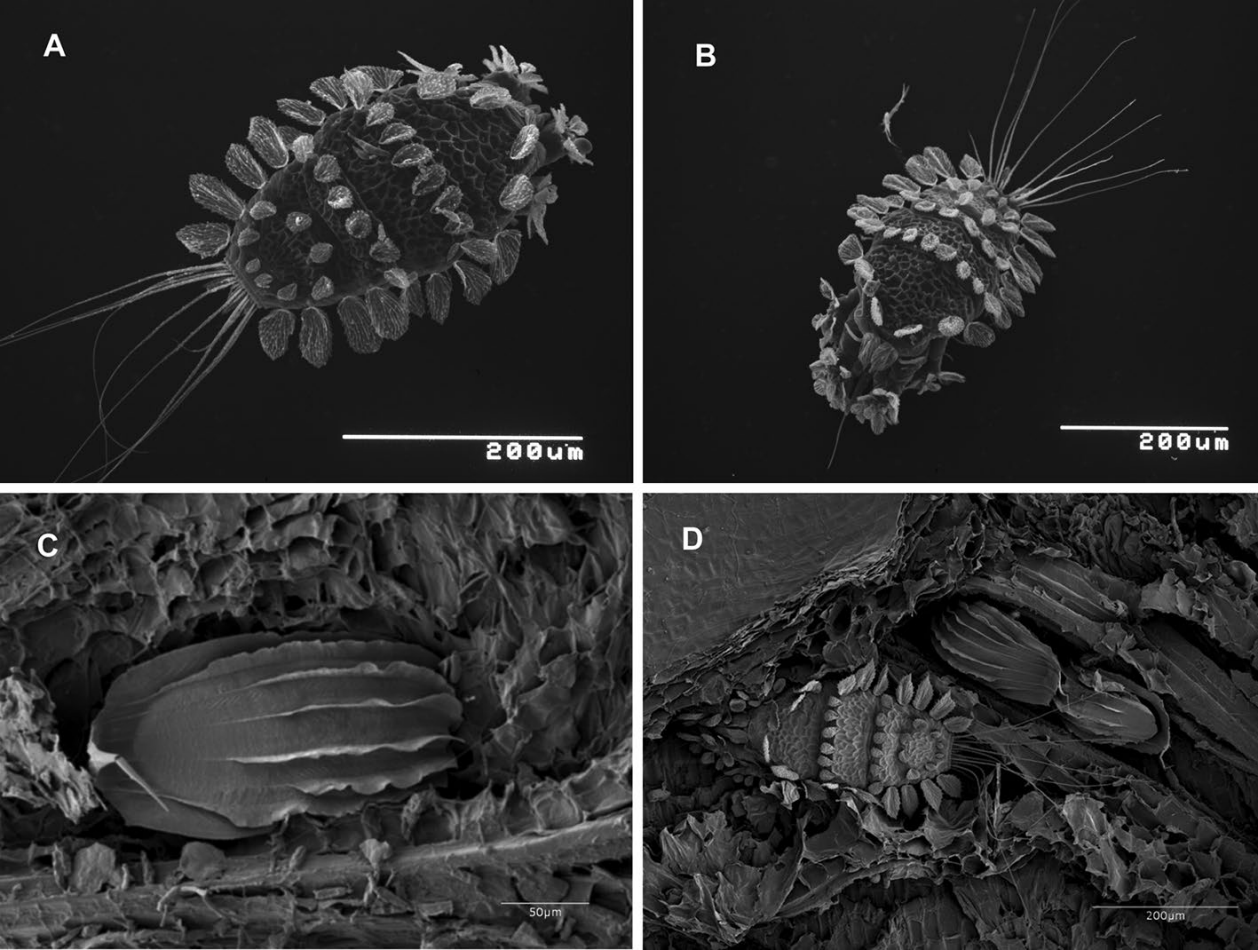
**a**, **b** The ornate dorsal setae of *Tuckerella japonica* and the mite’s ability to fold them against its body in tight areas. **c** Longitudinal ribbing on a *Tuckerella japonica* egg. **d** *Tuckerella japonica* female with two of her eggs

Like the Tenuipalpidae, motile stages of *T. japonica* are dorso-ventrally flattened. This enables the mite to fit into tight crevices formed by *C. sinensis* plants with outer bark splitting on the shoots (Fig. 1). The elaborate dorsal setae of the mite can fold down onto the body as it moves through these tight areas in the bark crevices (Fig. 2a, b). Although brightly colored, the mites can be overlooked on *Camellia* and other infested plant species.

The red colored egg of *T. japonica* is ribbed and relatively large, almost half the length of the adult female’s body (Fig. 2c, d). Eggs were deposited either in depressions within areas of split bark or other protected areas next to exposed periderm where the mites feed. The first two pairs of legs were observed in a video recording being used to pull the female forward and away from an emerging egg while ovipositing (Electronic Supplement 1).

### External morphology of the mouthparts of *Tuckerella japonica*

**Fig. 3:**
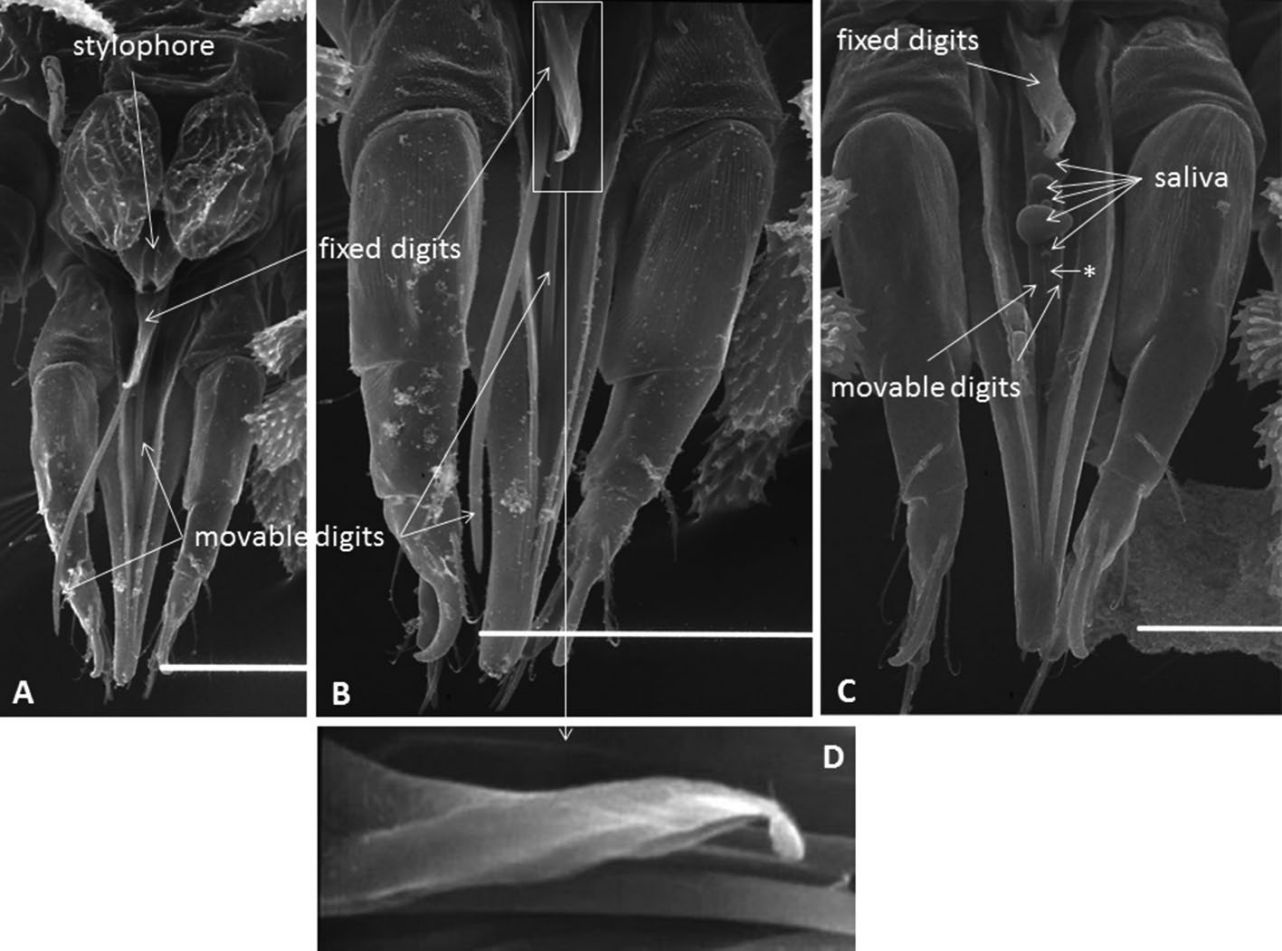
**a**–**c** The infracapitulum including the beak-like extension between the palpi, rostral gutter, stylophore, and the fixed and movable digits (= stylets) of *Tuckerella japonica*. Suspected saliva bubbles appear to come out from the dorsal side of the labrum. A thin fissure could be observed between the stylets (indicated with an asterisk). **d** Lateral view of the fixed digits

The external morphology of the mouthparts of *T. japonica* is similar to that of false and true spider mites, displaying a highly modified infracapitulum and chelicerae (Nuzzaci and de Lillo 1989, 1991a, b; de Lillo et al. 2001; Beard et al. 2012; Alberti and Kitajima 2014). The infracapitulum has a beak-like shape, such as in tenuipalpids, which is quite elongated in its distal part, projecting between the palps, and quite enlarged in its proximal part where it arises from the palp coxae (Fig. 3a–c). The paired movable digits are stylet-like, deeply located within the rostral gutter and kept well separated in this tract by the labrum (Fig. 3a–c). The fixed digits are located on the dorsal side, at the base of the infracapitulum, always between the vertical walls of the rostral gutter (Fig. 3b, c). This gutter is a dorsal, median and longitudinal deep groove formed on the dorsal sides of the infracapitulum. The rostral gutter guides and supports the stylets and the fixed digits during feeding (Nuzzaci and de Lillo 1989, 1991a). The location of the stylophore is shown in Fig. 3a.

**Fig. 4:**
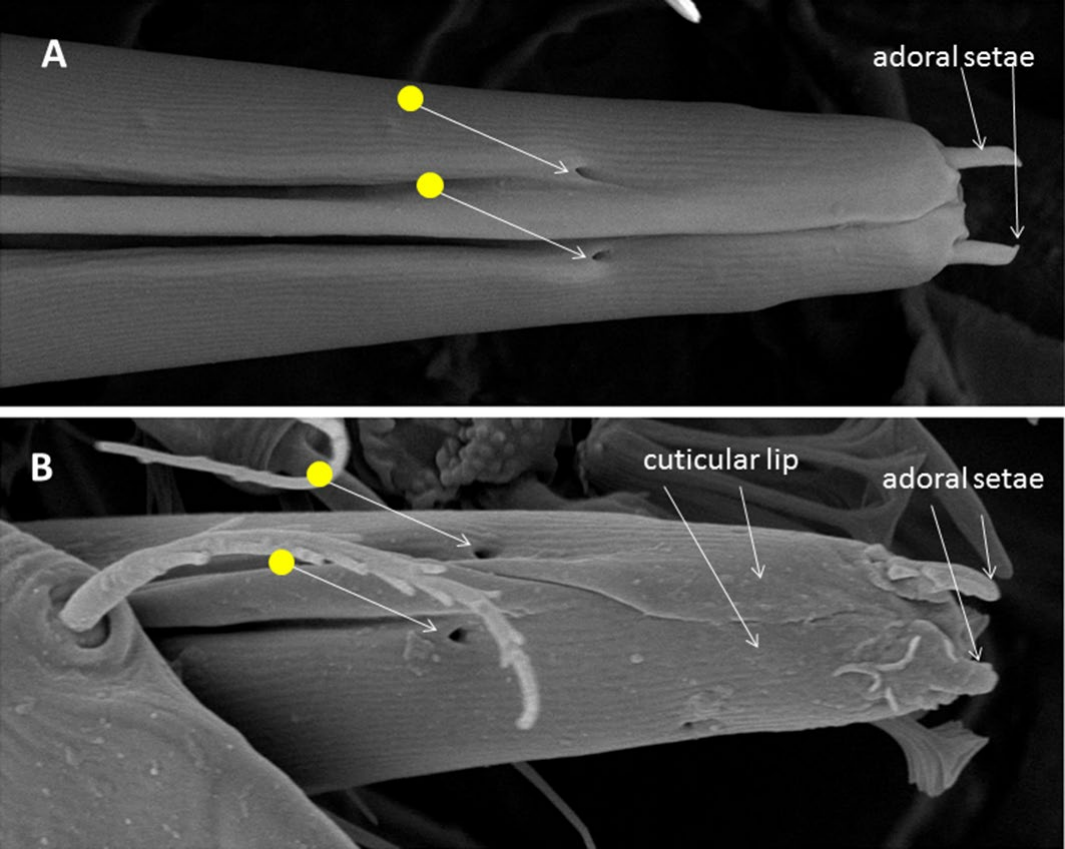
**a** The cuticular lips of *Tuckerella japonica*, produced dorsally by the lateral lips, appear to overlap on the distal part of the subcapitulum, whereas in **b** they appear paired and overlapping differently

One pair of adoral setae, with mechanoreceptive arrangement in other tetranychoids (Nuzzaci and de Lillo 1989, 1991a, b), and pits are evident at the distal end of the infracapitulum (Fig. 4). The adoral setae flank the opening at the distal tip of the infracapitulum through which the stylets are protracted. These setae are in contact with the surface of the plant at the beginning of feeding. The pores (yellow 2 circles with white arrows in Fig. 4) are on the dorsal sides of the infracapitulum or cuticular lips (Andre and Remacle 1984, for spider mites). These are not present on the infracapitulum of false and true spider mites and cannot be interpreted without a fine morphological study. They may be sensillar pits whose cuticular apparatus is undetectable by SEM.

**Fig. 5:**
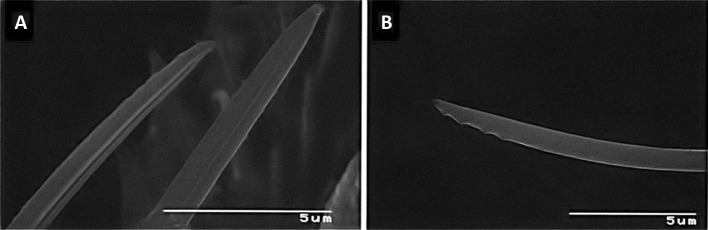
Distal parts of single, curved and serrated stylets of *Tuckerella japonica*

The stylets are similar to those of tenuipalpids and their tips are serrated on the antiaxial side (Fig. 5). When compared with true and false spider mites, each stylet can move back and forth in separate stylet channels located at the bottom of the rostral gutter and at the level of the labrum. When the stylets are protracted through the reinforced tip of the infracapitulum, they are forced to interlock with each other, probably by means of a tongue and groove articulation present on the paraxial sides of the stylets. When the mite is not feeding, the stylets lie on or within the rostral gutter (Fig. 3a–c). The stylets are involved in piercing plant tissues and injecting saliva into the plant cells to allow pre-oral digestion.

**Fig. 6:**
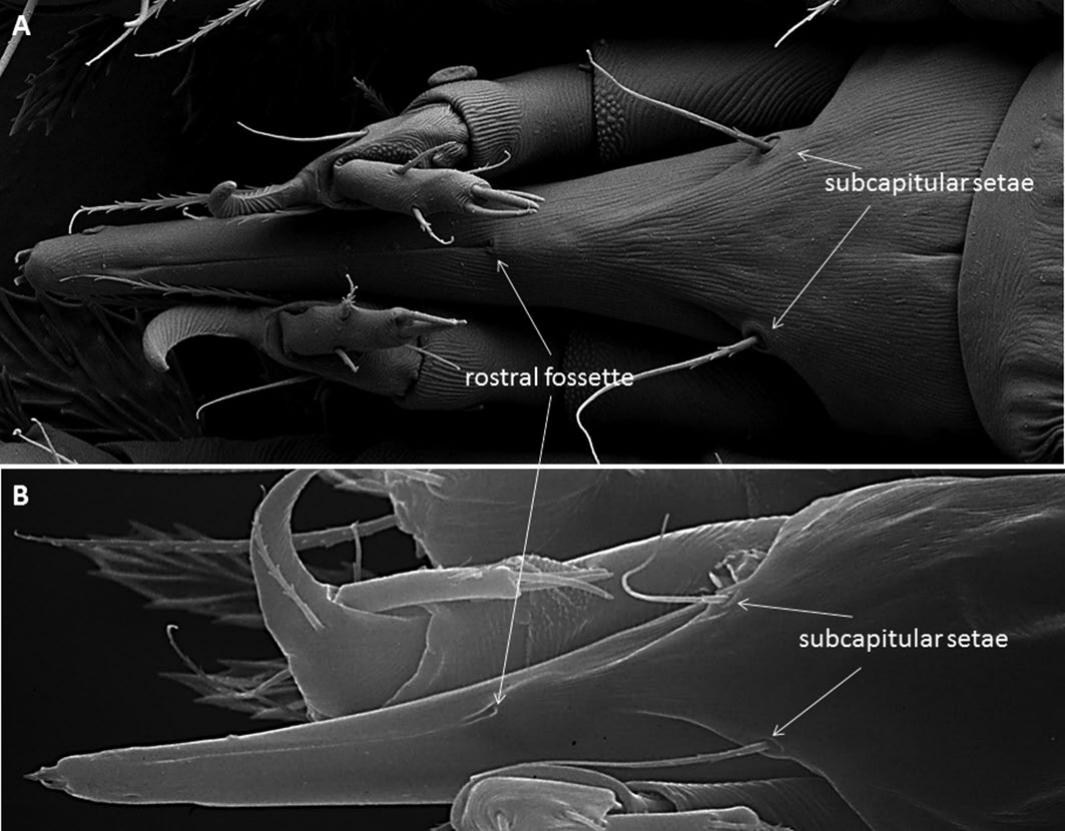
Ventral view of the infracapitulum of *Tuckerella japonica* showing the inferior oral commissure, the subcapitular setae and the enlarged basal part housing the pharynx

The beak-like infracapitulum of *T. japonica* contains a pharynx (observed on slide-mounted mites) and shows the rostral fossette, on its ventral side, suggesting the presence of the inferior oral commissure (Fig. 6).

### Feeding by *Tuckerella japonica on Camellia sinensis* stems

As in other tetranychoids, the stylets are forced to be interlocked as they pass through the reinforced distal tip of the infracapitulum when pressed on the feeding site and are held in place by the combined action of the rostral gutter, the stylet channels and labrum (Fig. 4) (Nuzzaci and de Lillo 1991a; de Lillo et al. 2001; Beard et al. 2012). The two stylets pierce plant tissues and inject saliva into the plant cells to allow pre-oral digestion (Nuzzaci and de Lillo 1989, 1991a). Pre-digested liquefied plant material is then drawn back into the gut of the mite through the pre-oral food channel.

*Tuckerella japonica* injects saliva resulting in cell wall disruption and collapsed cells and, in older tissue, induces hyperplasia occurring in the cortical layer of cells (Achor et al. 2017). As with other studied tetranychoids, the stylets are retracted, the infracapitular tip remains pressed on the wound and pre-digested liquefied cell contents are drawn back into the gut through the pre-oral food channel.

Protracted stylets were examined in slide-mounted specimens of *T. japonica*, and 9/10 had protracted, interlocked stylets that were curved medially. In contrast, the protracted stylets of *Brevipalpus* spp. consistently appeared straight in 11 slide-mounted specimens collected from *C. sinensis* as did those shown in LTSEM photographs of other *Brevipalpus* and *Raoiella* species (Ochoa et al. 2011; Beard et al. 2012; Alberti and Kitajima 2014).

The two stylets are stout compared to spider mite stylets and the tips are serrated and curved medially both individually and when combined (Fig. 5). This may be related to the different tissues which can be pierced by the two different taxa.

Since the mite’s stylets are flexible, their direction into pierced tissues could vary with each re-entry from the same puncture site thus allowing saliva to be introduced to more cortical tissues. It may account, in part, for the limited number of observed feeding holes on the exposed surface of periderm tissue infested with this mite. This ability to insert the stylets, withdraw them and insert them again through the same feeding hole may be similar to that of other tetranychoid mites (Summer and Stocking 1972; Hislop and Jeppson 1976; Beard et al. 2012). Curved stylets may not be more efficient and mites only require fewer access points to feed. Additional studies are needed to document stylet salivary tracts in periderm tissue of *C. sinensis*.

Achor et al. (2017) found the range of potential stylet depth into plant tissues for *T. japonica* was 92–150 µm for *T. japonica* as compared to 70–120 µm for several spider mite species (Avery and Briggs 1968; Tomczyk and Kropczynska 1985). The diameters of the two stylets combined ranged from 1.6 to 2.3 µm and were consistent with observed stylet punctures in the periderm (Achor et al. 2017). Unlike spider mites, *T. japonica* adults may have the potential to access outer phloem tissues with their saliva in 2-year-old stems of *C. sinensis* based on stem diameters, cell depth and stylet lengths (Achor et al. 2017) but this has to be verified experimentally.

*Tuckerella japonica* has been observed either attempting to penetrate or having penetrated green woody periderm tissue of *C. sinensis* with its stylets on several occasions. The mite places the tip of the infracapitulum against the plant surface of exposed periderm tissues with initial probing actions. In some instances, the mite moves around within limited areas as it probes followed by the mite attempting to penetrate the plant tissues with its paired stylets.

**Fig. 7:**
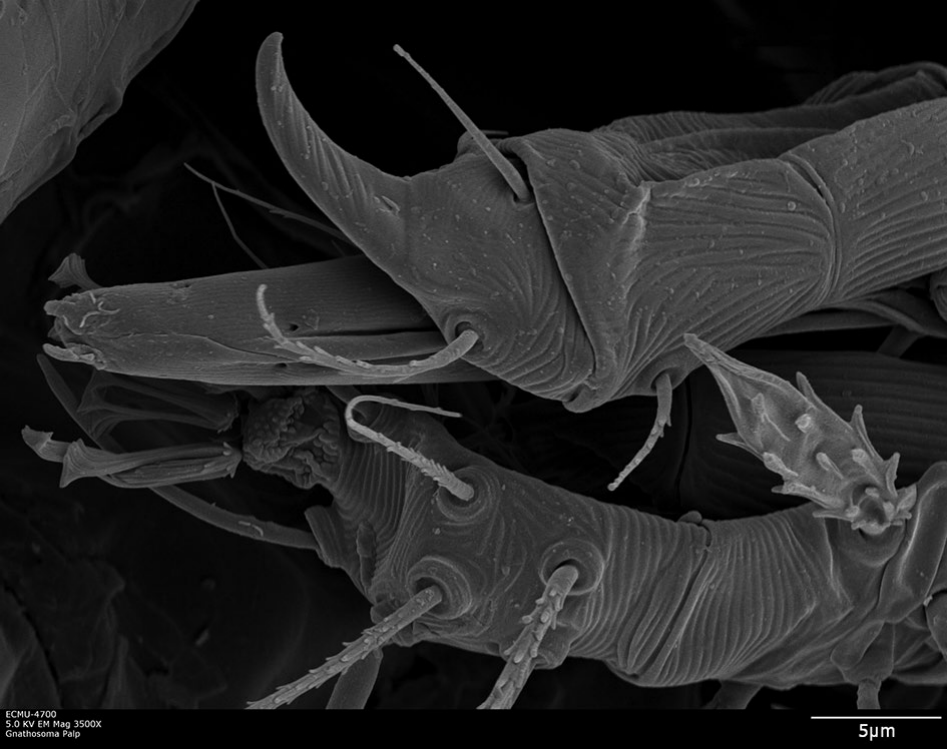
Palp claws of *Tuckerella japonica*

The beak-like part of the infracapitulum has been observed to bend as much as 90° downward to the surface of the periderm and the mite arches its body between legs II and III as it attempts to penetrate the exposed green plant cells. The infracapitulum appears to provide a platform of support and a guide for the paired stylets. The tip of the infracapitulum has not been observed to penetrate into the plant tissues. *Tuckerella japonica* also uses the palp claws as supporting anchors during the process of penetrating plant tissues of *C. sinensis* (Fig. 7).

Considerable effort appears to occur as the mite penetrates the plant cells of the periderm and into the cortical cells below. Pumping action within the mite’s body between legs I and II has been observed as the pharynx draws up the digested liquefied plant cell contents through the food channel. One instance was observed where the mite appeared to be feeding with the infracapitulum extended straight in contact with the surface of the periderm and at a slight downward angle.

### Scanning electron microscopy (SEM) study of the feeding holes made by *Tuckerella japonica* in periderm tissues of *Camellia sinensis*

Examples of feeding holes into periderm tissue produced by *T. japonica* are shown by Achor et al. (2017). They differ from slit-like cuts in the surface of the periderm made by the mite’s palp claws which were observed a few times under LTSEM photographs (Fig. 7).

At least one feeding hole into exposed periderm tissues was established near where one or more eggs would subsequently be deposited. There were eight stem pieces observed with single feeding holes and one stem piece with two feeding holes. No eggs or immature were present on the other sampled pieces. Does the location of a deposited egg(s) provide an accessible food source into the cortical layer of tissues for the emerging larvae? This question arises if proximity of eggs and immatures to the feeding hole(s) is an aide to the larva being able to feed at some point after eclosion. *Tuckerella japonica* larvae or nymphs were only occasionally observed feeding on *C. sinensis* during this study. The stylets of the larval stage and one or more nymphal stages may not be sufficiently strong enough to penetrate the exposed and intact periderm of *C. sinensis*. This may be the same situation that occurs on twigs of *Cryptomeria japonica*.

### Distribution and behavior of *Tuckerella japonica* and their progeny on *Camellia* branches

**Fig. 8:**
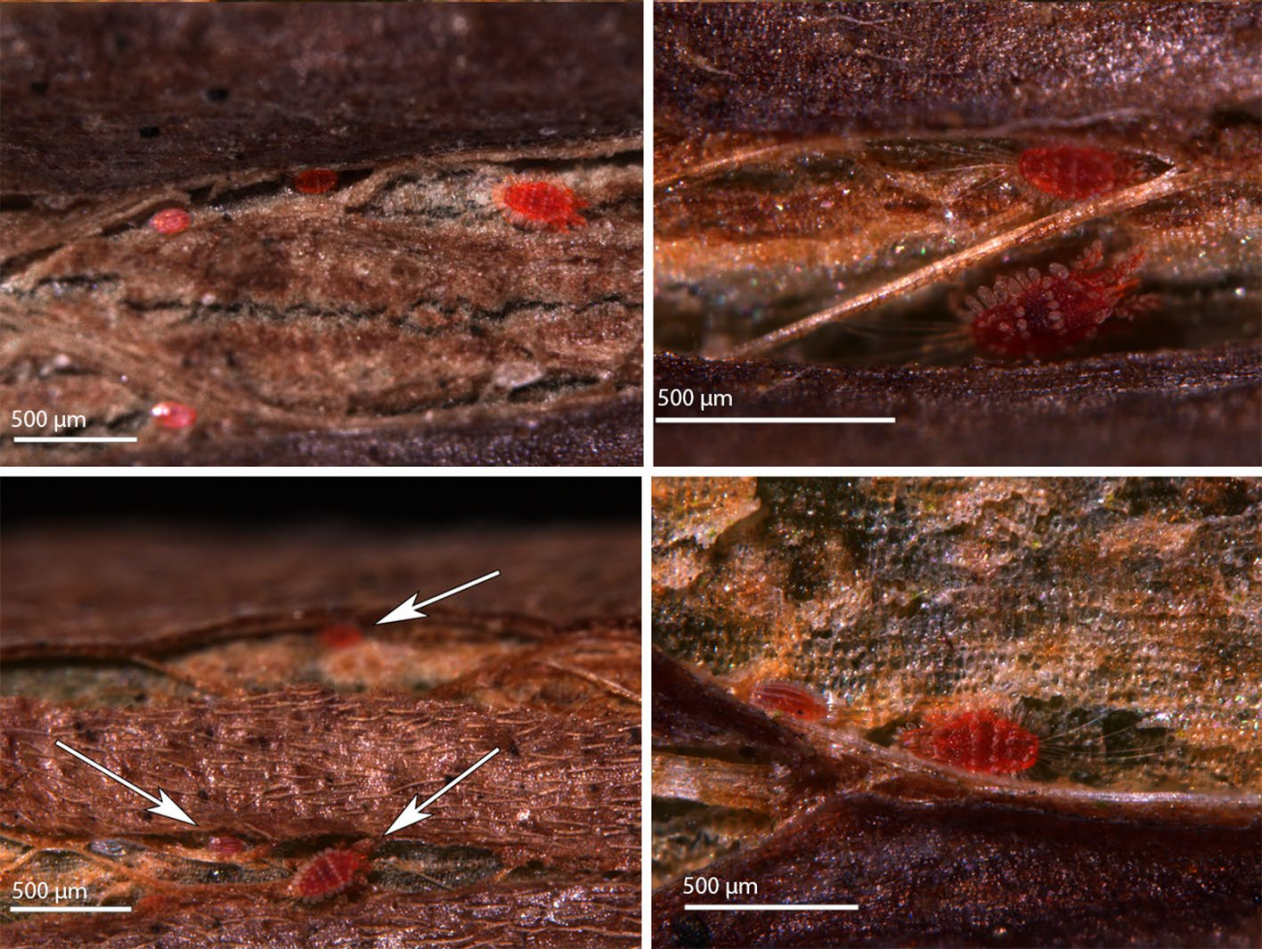
Four separate examples of aggregations of adult *Tuckerella japonica* with one or more eggs and/or immatures (indicated by white arrows) on stem sections of *Camellia japonica*

One or two eggs or an egg and/or immatures were frequently observed near an adult female at least 100× on stems on 12 May, 7 July, 27 October 2014, 21 September 2015, 25 May 2016 and 21 September 2016 (Figs. 2d, 8). A similar situation was shown by Zhang and Henderson (2013) where an adult and egg of *T. japonica* were found together on a twig of *Cryptomeria japonica*. The presence of one or more eggs or immatures with a female *T. japonica* on *C. sinensis* usually indicated the presence of one or more feeding holes based on SEM and LTSEM observations. The feeding holes on the stems were too small to be observed with a stereomicroscope. Often, it was difficult to see the periderm surface of older infestation sites of *T. japonica* due to accumulated debris including broken body parts or exuviae of this mite.

Areas of bark splitting on *C. sinensis* stems were shown to be the preferred habitat of *T. japonica* infesting *C. sinensis* bushes (Childers et al. 2016). Collyer (1969) reported *Tuckerella flabellifera* Miller favored rough areas and wounds on young wood and branches of its host plants with oviposition also observed at these locations.

**Fig. 9:**
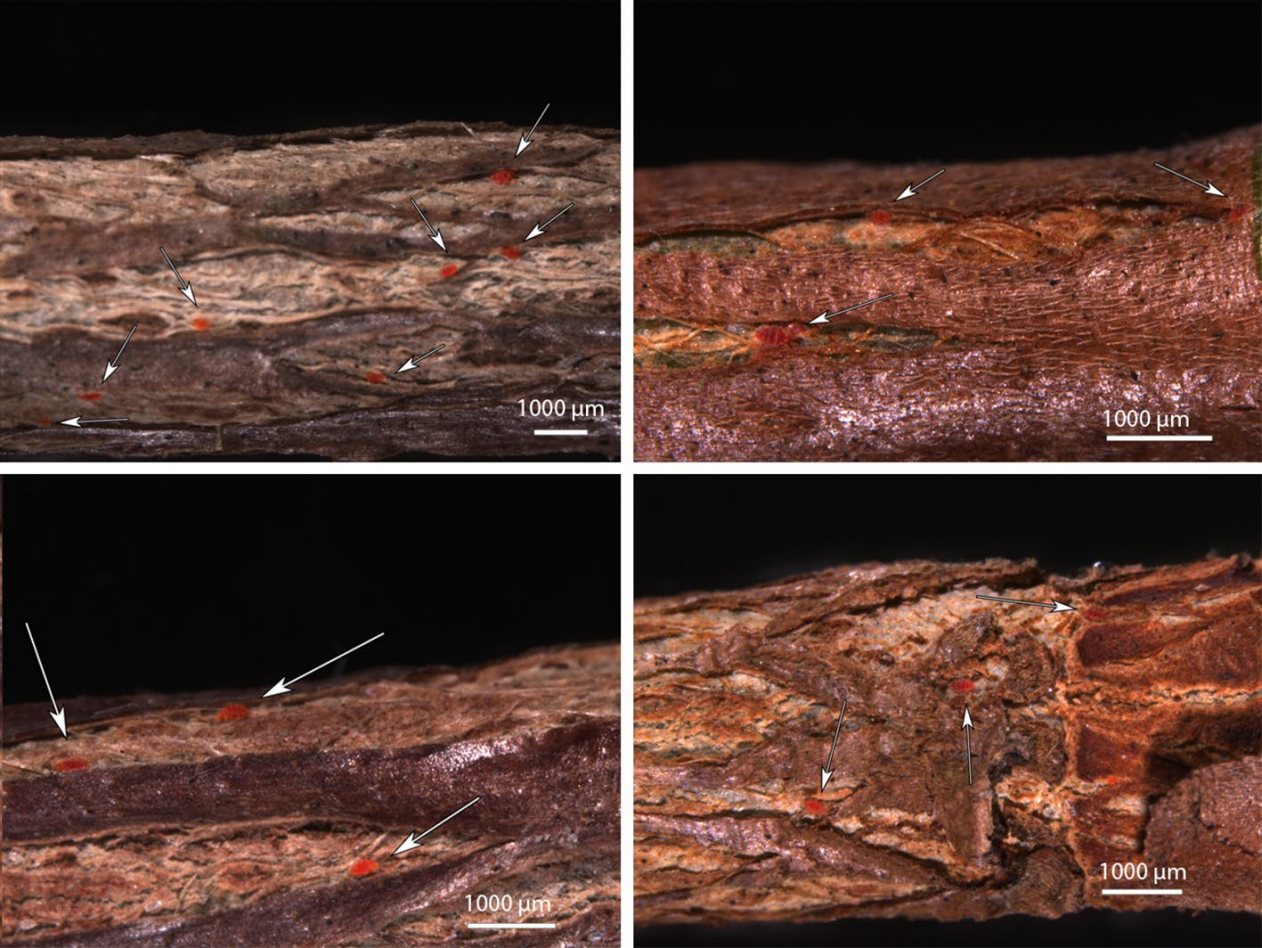
Four separate examples showing the spatial distribution of *Tuckerella japonica* motiles (indicated by white arrows) on periderm tissue of *Camellia sinensis* stems

*Tuckerella japonica* form clustered, family groups away from other tuckerellids on periderm tissues where bark splitting occurred. Examples of these spatially separated groups of females and their young were repeatedly observed on the various sample dates indicated. Four aggregations of adult *T. japonica* with one or more immatures are shown in Fig. 8. Figure 9 shows four separate instances of *T. japonica* females and/or immatures spatially separated on stems of *C. sinensis.* This distribution appears to be due to a combination of their territorial behavior and limited access to available feeding sites. As the branches age, there is a substantial reduction in bark splitting of the stems with no splitting occurring past 3 or 4 years (Childers et al. 2016).

Ewing (1922) first mentioned the caudal filaments of one tuckerellid species as being thrown forward like the feathers of a peacock which led to the common name for this group of mites. Use of caudal filaments as a defensive response was first reported by Collyer (1969) who observed these long terminal filaments of *T. flabellifera* to ‘thrash forward’ over the mite’s body when disturbed. This behavior in repelling predacious mites has since been observed with other tuckerellid species by Ochoa (1989) and Beard et al. (2013).

During the current research, one *T. japonica* female was observed protecting the area around her egg by aggressively warding off a tydeid mite and a *T. japonica* immature from her territory. In both instances, the female moved quickly towards the other mites. No indication of leg or caudal setal movements were noted. These observations were not video recorded. Similar instances were observed with this same behavior on two other occasions that involved an immature *T. japonica* and an unidentified acarid mite being aggressively charged by a female *T. japonica*.

One *T. japonica* female was video recorded beginning to deposit an egg near where she had been feeding. An immature *T. japonica* approached the female and she rapidly flipped her caudal setae forward and the immature responded in kind and then continued to pass with no further action by the female (Electronic Supplement 2). In another video not shown, possible kin recognition was recorded. One *T. japonica* female rapidly vibrated her caudal filaments, paused and then rapidly vibrated them again as a larva came close to the female. The larva immediately responded with the same sequence of rapid caudal filament vibrations. No action by the adult followed and the larva proceeded past the adult. It would appear that the actions identified the immature as her offspring. Kin recognition has been reported in predacious mites where they differentiated between their young and those not related (Schausberger and Croft 2001). Wilson (1987) defined kin recognition as the patterns and mechanisms entailed in the discrimination of kin from non-kin. Research is needed to identify these suspected behavioral signals and responses. All of these observed exchanges in signals occurred within a matter of seconds and single observations are worth reporting to provide leads for future research. Documentation of these events should be video recorded to allow follow-up analysis in detail.

Replicated samples of 244 cm of 1-, 2-, and 3+-year-old lengths of *C. sinensis* stems as well as green fruit and leaves were collected over 2 years at the Charleston Tea Plantation (Childers et al. 2016). Maximum numbers of *T. japonica* motiles collected from single 2-year-old stem samples occurred on 9 October 2013 and 27 October 2014 and their numbers were 247 and 111 mites, respectively (Childers et al. 2016). These counts equaled 2.1 and 0.9 motile stages per cm length of 2-year-old stems, respectively. The actual percentage of accessible feeding sites for this mite on a defined length of tea row or bush is unknown. In contrast, densities of *Eotetranychus sexmaculatus* (Riley), *Eutetranychus banksi* (McGregor), *Panonychus citri* (McGregor) (Tetranychidae) and *Brevipalpus* spp. (Tenuipalpidae) on citrus leaves in Florida have been observed to exceed 50 or more motile stages per leaf by the first author on numerous occasions. In these instances, leaf numbers and total surface area of available food are substantially greater for these mite species versus limited feeding sites within split bark areas that were available to *T. japonica* on *C. sinensis*.

Succulent green fruit of *C. sinensis* are not the preferred feeding site for *T. japonica* (Childers et al. 2016). The mite definitely feeds on the fruit but comparatively lower numbers were recorded on fruit between July and early September in South Carolina versus on stems of *C. sinensis*. This food source is temporary as small, immature fruit and darkening and hardening mature seed pods appear unsuitable as feeding sources (Childers et al. 2016). Thus, only a brief window of time is available for the mites to feed on this food source. Also, *Tuckerella* eggs were not observed on the smooth, exposed surfaces of developing fruit of *C. sinensis*.

### The potential of *Tuckerella japonica* as an economic pest of some *Camellia sinensis* seedlings or varietal plants

No studies have identified specific feeding sites of other tuckerellid species on their reported host plants. Feeding injury by multiple, overlapping generations of *T. japonica* on some C. *sinensis* seedling or varietal plants over time may result in long-term negative effects to plant growth, stem and shoot longevity, leaf production and/or reduced vigor or longevity of the entire plant. Insufficient data are available to determine whether tuckerellid species that occur on *Camellia* species are actionable pests requiring control.

Additional plantings of *C. sinensis*, with similar bark splitting characteristics and coming from identified varieties or seedling plants in different geographical locations, should be compared with the results reported in Childers et al. (2016) especially in *C. sinensis* plants with many young shoots per bush or linear meter of plant row.

### Sampling for tuckerellid mites

When sampling for populations of *T. japonica* and other tuckerellid species, key plant parts need to be sampled separately from leaves, twigs, fruit and stems on their identified hosts of economic importance over time and preferably at different locations. It is important to determine where and when tuckerellid mites are located on a plant and to identify precisely where they are feeding on those plants of economic importance supported with TEM and SEM data.

Replicated alcohol wash samples of specified plant parts are one accurate, effective, time-saving way to identify the general locations of tuckerellid species or most other mites on different varietal or seedling plants or the same plant species in different locations (Childers and Denmark 2011; Childers et al. 2016, 2017). Mature or young leaves, stems, and fruit could be sampled first. More specific locations on a particular plant could follow including: varietal differences, leaf, stem or woody stems, sepals, healthy versus damaged fruit from disease or mechanical injury, etc. Selection of plant parts to be sampled depends on the specific plant involved. However, it is not an effective method for recovering tuckerellid eggs. Once the adult and immature tuckerellids are located on a plant, then follow-up in the laboratory using a stereomicroscope should identify the location of the mite’s eggs. The alcohol combined with strong agitation during sampling has provided reliable results for collecting motile stages. This method allows for storage of the samples for short periods of time before processing.

Another sampling method could be used where alcohol is not available and where disposal issues arise. Washing mite-infested plant materials in a solution of bleach and detergent has been shown to be effective (de Lillo 2001; Monfreda et al. 2007). This method also can be used on undetached parts of some plants in order to determine their re-colonization. Once the primary feeding locations are determined within a cropping system then subsequent research can follow using microscopy, TEM or LTSEM to characterize feeding injury (Albrigo et al. 1983; Nuzzaci and de Lillo 1991a, b; Achor et al. 2017).

## Electronic supplementary material

Below is the link to the electronic supplementary material.

Video fragment showing a female Tuckerella japonica using her first two pair of legs to pull herself forward and away from an emerging egg during oviposition (MP4 10119 kb)

Video fragment showing a female Tuckerella japonica flipping her caudal setae forward while ovipositing as an immature T. japonica approached her. The immature responded with the same flipping of the caudal setae forward and then passed with no further response from the female (MP4 22934 kb)
